# Predation selection impacts social information use in guppies (*Poecilia reticulata*)

**DOI:** 10.1093/beheco/arag036

**Published:** 2026-04-22

**Authors:** Catarina Vila-Pouca, Hannah De Waele, Lisa Nijhuis, Regina Vega-Trejo, Marc Naguib, Alexander Kotrschal

**Affiliations:** CEFE, Univ Montpellier, CNRS, EPHE, IRD, 1919 Route de Mende, 34293 Montpellier, France; Behavioural Ecology Group, Wageningen University & Research, De Elsy 1, Wageningen 6708 WD, The Netherlands; Behavioural Ecology Group, Wageningen University & Research, De Elsy 1, Wageningen 6708 WD, The Netherlands; Department of Biology, Edward Grey Institute, University of Oxford, Zoology Research and Administration Building11a, Mansfield Road, Oxford OX1 3SZ, United Kingdom; Behavioural Ecology Group, Wageningen University & Research, De Elsy 1, Wageningen 6708 WD, The Netherlands; Behavioural Ecology Group, Wageningen University & Research, De Elsy 1, Wageningen 6708 WD, The Netherlands

**Keywords:** artificial selection, predation, cognitive evolution, guppies, social information use, social learning

## Abstract

Social information use helps animals to rapidly acquire biologically relevant information, but it can increase competition or be erroneous. The “costly information’ hypothesis proposes that animals especially rely on social information in high-risk contexts such as under predation pressure. However, whether predation drives the evolution of social learning remains enigmatic. Here we use artificial selection lines of Trinidadian guppies (*Poecilia reticulata*) that had been selected for predation survival for 3 generations (and control lines that were visually and olfactory exposed to predation but in a secured enclosure) to test how predation selects for social information use. In our study, social information use was assessed in a low-risk foraging context, which allows us to isolate evolved differences from acute responses under high-risk. Individuals observed a demonstrator on a screen solving a color association task, correctly or incorrectly, before attempting the task themselves. We show that individuals from both treatments learned the task, but only animals from the control lines relied on the use of social information during the process, while animals from the predation lines prioritized private information. Our results challenge the assumptions that social information use has adaptive benefits under increased predation risk. On the contrary, we propose that under high-predation certain social learning mechanisms may incur excessive costs due to, for instance, extensive acquisition time. We suggest that in a high-predation environment, disregarding social information and relying on personal information can be an adaptive strategy.

## Teaser

Animals may avoid social learning under high predation risk, relying more on personal experience to save time and reduce danger. This study on guppies shows that predation may favor individual learning as a better strategy, even in a low-risk foraging context.

## Introduction

Social learning, or the ability to learn from others (Heyes 1994; Box 2012), offers additional information to individual (or asocial) learning by allowing an animal to acquire biologically important information efficiently and to decrease risk. This type of learning can be advantageous when asocial learning diverts time away from other activities that enhance fitness, and can pose risks (Palameta and Lefebvre 1988). For example, prolonged time spent learning can be used to forage or find a reproductive mate; similarly, asocial learning may increase predation risks. Social learning is therefore often considered to be more efficient when acquiring locally adaptive behaviors, as it allows naïve individuals to adopt behaviors from more experienced conspecifics without carrying the costs associated with trial-and-error learning processes (Boyd and Richerson 1988; Brown and Laland 2003; Hoppitt and Laland 2013). For example, by copying others, naïve individuals can quickly learn the location of essential resources such as food and water (Galef and Giraldeau 2001), identify strategies to evade predators (Griffin 2004), or learn with whom to mate (White 2004). Consequently, the ability to learn socially may increase an individual's survival and reproductive success under certain conditions.

However, social learning can also carry costs, as it can lead to increased competition for the same resources or it can make animals act on outdated, irrelevant, or maladaptive information (Boyd and Richerson 1988; Giraldeau et al. 2002; Rieucau and Giraldeau 2011). By weighing the costs and benefits of social learning, individuals and populations may exhibit different propensities for social learning (Laland 2004; Kendal et al. 2009a; Heyes and Pearce 2015). The inclination to rely on social learning aligns with the “costly information hypothesis” (Boyd and Richerson 1988). According to this hypothesis, animals are more likely to depend on social information in high-risk situations where the costs of asocial learning are typically greater. Indeed, this hypothesis is supported by empirical evidence, demonstrating that individuals are more likely to use social information for more challenging tasks and/or when the costs of asocial learning are increased (Kendal et al. 2009b; Laland et al. 2011; Morgan et al. 2012; Baracchi et al. 2018). Such costs of asocial learning may stem from the risk of individual exploration (Rieucau and Giraldeau 2011), as in environments with intense predation pressure, where venturing alone is typically dangerous. Animals are therefore expected to show a greater reliance on social learning under such conditions as social learning allows individuals to acquire information rapidly and efficiently from more knowledgeable conspecifics (Brown and Laland 2003). For example, 3-spined sticklebacks (*Gasterosteus aculeatus*), that are at a higher risk of predation compared with 9-spined sticklebacks (*Pungitius pungitius*), show comparably higher susceptibility to social influence when selecting a feeding site (Coolen et al. 2003). Numerous indirect lines of evidence suggest that predation is linked to increased use of social information, especially for anti-predator behaviors (Suboski and Templeton 1989; Chivers and Smith 1995; Kelley and Magurran 2003; Griffin 2004; Mineka and Cook 2013).

Social learning is taxonomically widespread, from mammals to insects (Palameta and Lefebvre 1988; Heyes and Galef 1996), including many fish species (Brown and Laland 2003). Guppies (*Poecilia reticulata*) are especially well studied in several contexts: they use social learning to evade predators (Kelley et al. 2003), copy mate choice decisions (Dugatkin and Godin 1992), and learn foraging routes (Laland and Williams 1998; Swaney et al. 2001). Throughout the years, the Trinidadian guppy has also become a model system in ecology and evolution. In particular, researchers have taken advantage of the guppy system to explore the impact of predation pressure on the evolution of various life-history, morphological, and behavioral traits (reviewed by Magurran 2005). This is because guppies inhabit parallel streams that naturally exhibit a range of predation pressure (Reznick et al. 2001; Magurran 2005): downstream areas are typically home to several predator species that prey on adult guppies (high-predation environments), while waterfalls block access of most predators to upstream regions that therefore have few to no such predators (low-predation environments). These replicated predation differences provide the opportunity to investigate how predation pressure influences the evolution of social behavior and learning. For example, when guppies from low-predation environments are exposed to a predator model while in the presence of guppies from high-predation environments, they improve their anti-predator behavior through the association with these more experienced conspecifics (Kelley and Magurran 2003). Similarly, another study found population differences between high- and low-predation populations in the Aripo river, with fish from low-predation environments avoiding demonstrated feeding locations, while those from high-predation locations seemed to prefer them (Chouinard-Thuly and Reader 2019). These and related studies (Seghers 1974; Farr 1975; Heathcote et al. 2017) have allowed to examine the interplay between social behavior and predation risk.

However, studying the effect of predation on social information use through the comparison of natural populations introduces the challenge of varying ecological characteristics in addition to predation pressure differences. For instance, when comparing wild populations of guppies, streams with high-predation pressure also tend to have lower species richness, lower conspecific density, wider channels, more open canopies, and higher levels of primary productivity (Hawkins et al. 1982; Reznick and Endler 1982; Power 1984; Feminella et al. 1989; Magurran 2005). Hence, when comparing the social information use of animals from low and high-predation populations, the effect of predation is inherently confounded by other ecological factors that may also impact information use strategies. Conspecific density, for example, likely impacts social information use as guppies reared at low densities typically show a higher shoaling tendency and find food more efficiently than guppies reared at higher densities in a maze trial with trained demonstrators (Chapman et al. 2008). Therefore, while these populations with varying predation pressures provided insights into predator-driven social information use and social learning, the causal impact of predation pressure on the evolution of social information use is still unclear. Therefore, we tested fish with divergent predation histories in a low-risk environment using a demonstrator-observer paradigm. By utilizing recently established selection lines for predation survival in guppies, we experimentally assessed, for the first time to our knowledge, how predation pressure directly selects on the use of social information.

We compared the performance of fish from those predation selection lines to that of control lines in a social learning task. We gave animals access to either correct or incorrect social information while learning a color association task. If fish are socially learning the task, they should prefer the color chosen by the demonstrator they observed. As the task was low-risk and involved predator-naïve offspring, variation in social information use in our study would reflect evolved differences or plasticity in information-use strategies, rather than acute responses to predation. In line with the costly information hypothesis and empirical studies investigating social learning and predation pressure (Suboski and Templeton 1989; Chivers and Smith 1995; Kelley and Magurran 2003; Griffin 2004; Mineka and Cook 2013), we predicted that predation would select for increased social information use. Thus, we expected the effect of the available social information on learning performance to be stronger for guppies from the predation-selected lines compared with those from the control lines.

## Methods

### Selection experiment

We examined the selective effect of direct adult predation on social information use by comparing experimental lines of fish (*Poecilia reticulata*) subjected to divergent predation pressures over 3 generations.

We used laboratory descendants of Trinidadian guppies collected from high-predation populations from the Quare River in 2005. Stock populations were kept in several large tanks for 14 years, without predators, at Trondheim University. In 2010, 150 animals were brought from Trondheim to Stockholm to start an initial stock population (Vega-Trejo et al. 2022; De Waele et al. 2025). In 2018, more animals were brought from Trondheim to Stockholm to set up 110 breeding pairs (F0), from which juveniles for 3 replicates were produced (F1). These juveniles were supplemented with 100 juveniles from the initial stock population (F1). While our population originated from a high-predation locality, we assume that after 14 years of relaxed selection with a species known for its fast evolution (Reznick et al. 2001), we were starting with a low-predation population.

The selection experiment was designed to lead to 2 distinct groups of guppies based on their survival of a predator (*Crenicichla alta*). In the first group (‘Predation” lines), we established 3 replicate lines of guppies that underwent continuous selection for survival under adult predation (until the number of surviving fish reached our target; survival rate of approximately 19%) for 3 subsequent generations. The second group (‘Control” lines) served as a control consisting of 3 replicate lines of guppies exposed to sensory cues from a predator but never facing actual predation. Once each selection experiment was completed, males and females from the same treatment and replicate were paired up together to produce the next generation. Once matured, the progeny was subjected to the same selection procedure. We did this for 3 continuous selection events, and thus 3 generations. We here assess social information use in the offspring of the third-generation fish (F4), which were kept under the same conditions (ie no exposure to predation either directly, nor sensory cues). For further details on the selection procedure, see (Vega-Trejo et al. 2022; De Waele et al. 2025).

### Social learning task

#### Individuals

Individuals used in the learning task were 50 adult females at an approximate age of 12 months. Of these, 26 individuals were from the predation-selection treatment (of which *n* = 10, 8, and 8 from replicates 1, 2, and 3, respectively) and 24 were from the control treatment (*n* = 9, 7, and 8 from replicates 1, 2, and 3, respectively). Only females were tested, as males are often difficult to motivate with a food reward (Fuss et al. 2021; Vila-Poucaet al. 2022a).

#### Testing apparatus

**Figure 1:**
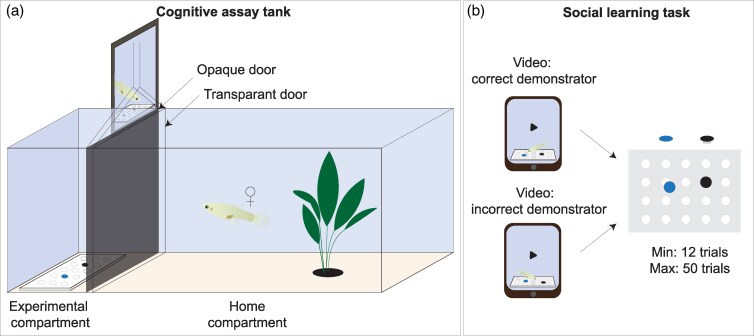
a) Experimental tank used to compare social learning in predation-selected and control guppies. The tank consisted of a home compartment and an experimental compartment separated by an opaque and transparent door. b) Schematic representation of the social learning task, where fish were shown a video of either a correct demonstrator or an incorrect demonstrator after which they were given a choice between 2 colored discs, 1 blue and 1 black, both concealing a food reward in the hole underneath them. The negative stimulus (here represented in black) was unmovable.

During the experiment, the fish were housed in individual tanks. The tanks consisted of a home compartment (25 × 15 cm) and an experimental compartment (15 × 15 cm). The 2 compartments were separated by 2 guillotine doors, 1 transparent and 1 opaque, making the experimental compartment only accessible during experimental trials. The rest of the time the fish resided in the home compartment with visual contact to fish in neighboring tanks for social enrichment. To prevent nonintended social learning effects, we placed opaque dividers between experimental compartments of neighboring tanks. Inside each experimental compartment, a white plate (14 × 10 cm) with 20 circular holes (5 mm deep, 10 mm diameter), was placed. These plates allowed a food reward to be concealed within one of the holes, which could be covered by a colored disk. To retrieve the food reward, the colored disk had to be dislodged (Fig. 1). The fish were randomly distributed across 50 tanks, and the experimenters were blind to the treatment of each animal.

#### Pretraining

During pretraining, fish learned to dislodge a green disc to access a food reward (1 frozen adult *Artemia*) hidden in the middle hole. The trial started with the opening of the opaque door; 5 s later, we opened the transparent door. The fish could then voluntarily enter the training compartment and find the food reward. During the first trials, the disc only partially covered the hole, leaving the reward exposed. We then trained the fish to dislodge the green disc by successively moving the disc from partially to fully covering the hole. Before starting the social learning task, fish were given 3 trials, where the green disc fully covered the hole. All females succeeded in dislodging the disc in all 3 trials and continued to the experiment.

#### Social learning task

The social learning task consisted of the fish being shown a video of a demonstrator at each trial, after which they performed a well-established color association task (Buechel et al. 2018; Vila-Pouca et al. 2022b; Vila-Pouca et al. 2025), using black and blue as stimulus colors. Fish from the 2 predation-selection groups (predation or control lines) randomly received 1 of 2 demonstrator group treatments: correct or incorrect demonstrator, balanced across treatments and replicates. A trial started when the opaque door was opened, allowing the fish to view the video of the demonstrator on a tablet (Samsung Galaxy tab A7 lite) inside a waterproof sleeve that was placed inside the experimental compartment, behind the opaque door. This made the video perspective for the fish in the home compartment resemble the presence of a demonstrator in the experimental compartment. In the video presenting a correct demonstrator, the demonstrator took the food reward from the color assigned as “correct” for the observer fish. With an incorrect demonstrator, the demonstrator would collect the food reward from the colored disc assigned as “incorrect” for the observer. After the video finished, the opaque door was closed and the tablet taken out of the tank. 20 s later the opaque door was opened again, followed by the transparent door 5 seconds later, to start the color association task for the focal fish (see below). Fish in general (Rowland et al. 1995, 1996; Körner and Parzefall 1997; Balshine-Earn and Lotem 1998; Clark and Stephenson 1999; Rosenthal 1999), and guppies (Kodric-Brown and Nicoletto 1997; Nicoletto and Kodric-Brown 1999; Kodric-Brown and Nicoletto 2001) in particular, respond well to video playbacks. Additionally, videos have the advantage of standardizing visual stimuli, enabling the presentation of identical stimuli to different individuals (Nicoletto and Kodric-Brown 1999). The videos of the fish had been recorded prior to the experiment. Both demonstrations with black and blue were recorded for 2 different demonstrator fish individuals, to prevent individual preferences (Lachlan et al. 1998). The videos were recorded on a Canon Eos 250D, with a 18 to 55 mm lens (F5.0; ISO 3200; 1/200). The videos were adjusted in size using Adobe Premiere Pro, ensuring the recorded fish's proportions to appear life-sized.

During the color association task, the fish had the choice between 2 colored discs (blue and black), both concealing a food reward (to ensure fish could not learn based on olfactory cues). The spatial positioning of the colored discs corresponded to those displayed on the video the fish had previously seen. Only one of the discs could be dislodged, while the other disc was loosely held in place by a silicone stopper attached beneath the disc, which was not visible to the fish. Before the start of the experiment, fish were randomly assigned to either black or blue as the correct stimulus, balanced across treatments and replicates. Additionally, for each trial, we randomized the position (left or right) of the correct color, with no more than 2 consecutive trials in the same position, to avoid side biases. The first disc the fish touched was recorded as the fish's choice. The fish was given 1 min to dislodge the correct disc and eat the reward. When a fish chose the wrong disc, correction was allowed within 3 min. If the fish failed to correct its choice within 3 min, or when fish failed to make any choice within 1 min, we moved the rewarded disc 5 mm to the side during the trial to allow easier access to the food (using a transparent pipette). This ensured that all fish experienced the same number of food rewards and reinforced trials throughout the experiment, regardless of their initial choice. All fish ran 3 daily trials until they reached learning criterion (minimum 12 trials). The learning criterion consisted of 7 correct choices consecutively (significant according to a binomial probability). If a subject did not reach learning criterion within 50 trials, we concluded that they did not learn the task.

### Statistical analysis

Final sample sizes were: Control correct demonstrator *n* = 13; Control incorrect demonstrator *n* = 11; Predation correct demonstrator *n* = 13; Predation incorrect demonstrator *n* = 13. Statistical analyses were performed in R (version 4.3.2) (R Core Team 2022) using “lme4' (version 1.1 to 35.1) (Bates et al. 2015). We analysed both initial-trial performance and learning rates over different trial windows because these complementary measures capture distinct aspects of social information use: the first trial reflects immediate copying behavior, whereas learning curves across multiple trials incorporate individual learning processes and allow us to assess how quickly fish adjusted when demonstrator information was incorrect. We compared the performance of predation and control guppies in: (i) Number of fish that learnt the task (1 = learnt; 0 = failed) using a generalized linear model (GLM, binomial distribution) with predation-selection treatment, demonstrator correctness, replicate, color, and the interaction of predation-selection treatment × demonstrator correctness as potential predictor variables; all fish were included in model 1 (*n* = 50); (ii) Number of trials to reach learning criterion (GLM, Poisson distribution) with predation-selection treatment, demonstrator correctness, replicate, color, and the interaction of predation-selection treatment × demonstrator correctness as potential predictor variables; only fish that learnt were included in model 2 (*n* = 38); (iii) Choice in the first trial (1 = correct; 0 = incorrect; GLM, binomial distribution) with predation-selection treatment, demonstrator correctness, replicate, color, and the interaction of predation-selection treatment × demonstrator correctness as potential predictor variables; (iv) Learning rate of the initial 15 trials, ie probability of success per trial (1 = correct; 0 = incorrect) using a generalized linear mixed model (GLMM, binomial) with trial number, predation-selection treatment, demonstrator correctness, replicate, color, and the interaction of trial number × predation-selection treatment × demonstrator correctness as predictor variables and a random intercept and slope for fish identity, which accounts for the repeated observations of individual fish; (5) Learning rate across all trials (1 = correct; 0 = incorrect) using a binomial GLMM with trial number, predation-selection treatment, demonstrator correctness, replicate, color, and the interaction of trial number × predation-selection treatment × demonstrator correctness as predictor variables and a random intercept and slope for fish identity. Both learners and nonlearners were included in models 3, 4, and 5, however 5 fish were excluded from this part of the analysis because of repeated refusal to engage in the task (<30% recorded choices during training trials; models 3, 4, and 5 *n* = 45).

We note that, in all models, replicate was included as a fixed effect (and not as a random effect as per our nested design) because it only has 3 levels and our sample size was limited (Bolker et al. 2009; Oberpriller et al. 2022). For models with significant interaction terms, we computed estimated marginal means and conducted pairwise comparisons between contrasts using the “emmeans” package (Lenth 2023), reporting rate ratios and 95% confidence intervals as effect sizes.

## Results

Of the 50 guppies tested, most learnt to associate a colored disc with a food reward in the social learning task within the given number of trials. The number of successful fish was independent of the predation-selection treatment and demonstrator correctness, with no significant interaction or main effects detected (Table 1; treatment × demonstrator: est = 18.13, SE = 2846.98, *Z* = 0.01, *P* = 0.99). We observed that 18 out of 26 (69.2%) fish in the predation treatment, and 20 out of 24 (83.3%) in the control treatment succeeded in the task (est = 0.32, SE = 0.97, *Z* = 0.33, *P* = 0.74). Similarly, fish with the incorrect versus correct demonstrators showed similar success rates (est = 0.87, SE = 1.01, *Z* = 0.86, *P* = 0.39). The number of successful fish was higher in fish where black was the rewarded color (est = −2.40, SE = 0.92, *Z* = −2.62, *P* = 0.009), but the potential influence of this difference on the results was ruled out by evenly distributing the colors among groups. We also found no differences in success rate between replicate lines (est = −0.67, SE = 0.54, *Z* = −1.32, *P* = 0.19).

**Table 1:** Outcomes of statistical models for the social learning task.

Factors	Estimate	Standard error	*Z* value	*P*-value
**Model 1: Proportion of fish learning association (N_ind_ = 50)**				
**C+, *n* = 13; C−, *n* = 11; P+, *n* = 13; P−, *n* = 13**				
Intercept	3.21	1.41	2.27	**0.02**
Predation-selection treatment	0.32	0.97	0.33	0.74
Color	−2.10	0.92	−2.62	**0.01**
Demonstrator correctness	0.87	1.01	0.86	0.39
Replicate	−0.67	0.54	−1.32	0.19
Treatment:Demonstrator	18.13	2846.98	0.01	0.99
**Model 2: Trials to association criterion (N_ind_ = 38)**				
**C+, *n* = 9; C−, *n* = 11; P+, *n* = 8; P−, *n* = 10**				
Intercept	2.41	0.13	19.24	**< 0.001**
Predation-selection treatment	−0.21	0.13	−1.63	0.10
Color	0.60	0.08	7.82	**< 0.001**
Demonstrator correctness	0.08	0.12	0.72	0.47
Replicate	0.08	0.05	1.79	0.07
Treatment:Demonstrator	0.48	0.16	3.03	**0.002**

N_ind_, number of individuals; C+, control fish with a correct demonstrator; C−, control fish with an incorrect demonstrator; P+, predation fish with a correct demonstrator; P−, predation fish with an incorrect demonstrator. Significant values are given in bold.

**Figure 2:**
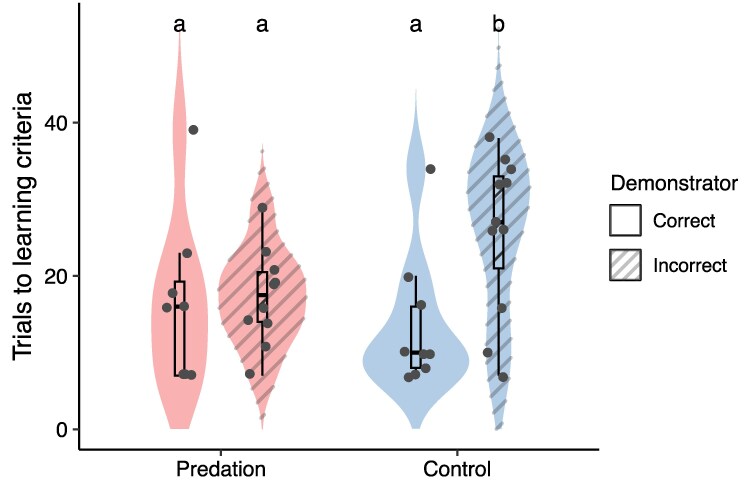
The number of trials predation-selected (left, red, *n* = 18) and control (right, blue, *n* = 20) guppies took to learn to associate a color with a food reward in dependance of correct (full) or incorrect (hatched) demonstrator information. Violin plots indicate the distribution of the data, while dots represent raw data points. Boxplots indicate the median and interquartile range (IQR), whiskers extend 1.5 × IQR.

Among the guppies that learned the task, when comparing the number of trials taken to learn, we found differences in social information use between predation-selection treatment groups (treatment × demonstrator correctness: est = 0.48, SE = 0.16, *Z* = 3.03, *P* = 0.002; Fig. 2). Control fish with an incorrect demonstrator were the slowest group to learn the task, needing significantly more trials to reach the learning criterion compared with all other groups. Specifically, they needed 1.8 times more trials than control fish with a correct demonstrator (‘emmeans” contrast: C+/C− rate ratio = 0.568, SE = 0.06, *z* = −5.20, *P* < 0.001). Moreover, control fish with an incorrect demonstrator needed 1.3 times more trials than predation fish with an incorrect demonstrator (P−/C− rate ratio = 0.759, SE = 0.08, *z* = −2.80, *P* = 0.027) and 1.4 times more trials than predation fish with a correct demonstrator (P+/C− rate ratio = 0.698, SE = 0.08, *z* = −3.37, *P* = 0.004). Control fish with a correct demonstrator did not significantly differ from both predation groups, but approached significance in taking 1.75 fewer trials to learn compared with predation fish with an incorrect demonstrator (C+/P− rate ratio = 0.75, SE = 0.09, *z* = −2.43, *P* = 0.07; P+/C+ rate ratio = 1.23, SE = 0.16, *z* = 1.63, *P* = 0.36). Finally, both predation groups took a similar number of trials to learn (P+/P− rate ratio = 0.920, SE = 0.11, *z* = −0.72, *P* = 0.89). These results indicate that fish from the control treatment relied on social information from the demonstrators, even when that information was incorrect, while predation fish scored intermediate and therefore likely relied on private information or were quicker in ignoring false information. We found no differences between replicate lines (est = 0.08, SE = 0.05, *Z* = 1.79, *P* = 0.07), but the number of trials to learning criterion was lower in fish that had black as the rewarded color (Table 1; est = 0.597, SE = 0.076, *Z* = 7.82, *P* < 0.001).

**Figure 3:**
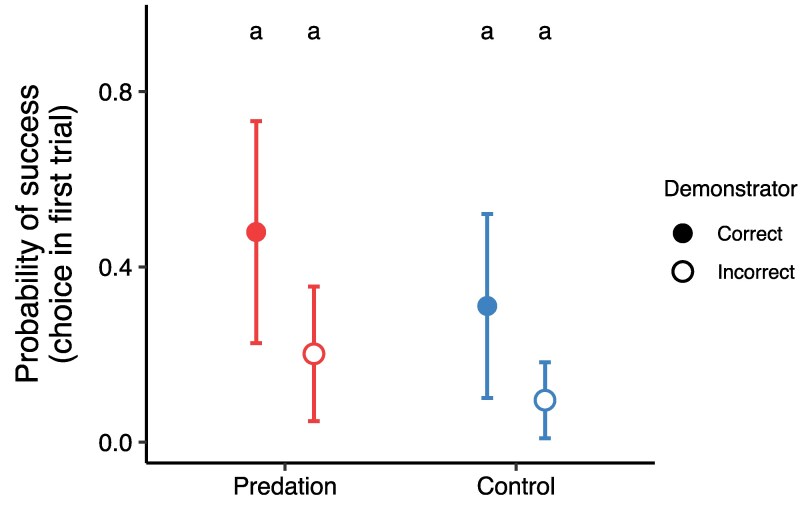
The probability of success in the first trial, where fish made a choice with no personal information about the task, for predation-selected (left, red) and control (right, blue) guppies in dependance of correct (full) or incorrect (open) demonstrator information. Points indicate estimated marginal means and whiskers the standard error, obtained from the interaction term treatment × demonstrator of model 3. The letters indicate that groups are not statistically different. Sample sizes: Predation correct demonstrator *n* = 11; Predation incorrect demonstrator *n* = 12; Control correct demonstrator *n* = 11; Control incorrect demonstrator *n* = 11.

We also examined choice in the very first trial of the task, to assess if fish were acting on social information and copying their demonstrators since they had no personal information about the task yet, and whether this varied with treatment and demonstrator group. We found no significant differences in proportion of success in the first trial between treatment and demonstrator groups (treatment × demonstrator: est = −0.16, SE = 1.79, *Z* = −0.09, *P* = 0.93), nor significant differences between treatment groups and demonstrator groups as main effects (both *P* > 0.05; detailed output in the supplemental information). However, there was a trend indicating that fish from both treatments with an incorrect demonstrator exhibited the lowest probability of success in this very first trial (Fig. 3). We found no differences in proportion of success in the first trial between replicates (est = 0.22, SE = 0.55, *Z* = 0.41, *P* = 0.68), while fish with black as the correct color had higher success (est = −4.37, SE = 1.27, *Z* = −3.43, *P* < 0.001).

**Figure 4:**
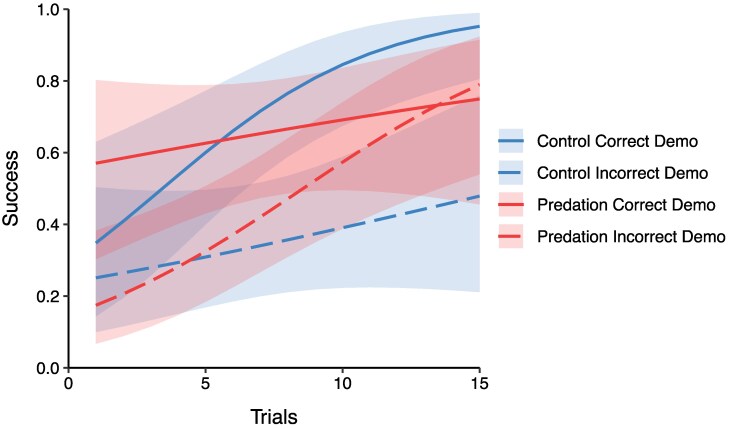
Probability of correct choice over the first 15 trials of female guppies with predation (red) and control (blue) treatments, exposed to the correct demonstrator (full line), and incorrect demonstrator (dashed line). The coloured line shows predictedmodel outputs and shaded areas indicate 95% confidence intervals.

When comparing learning rates at the start of the task (over the first 15 trials, to examine if certain treatment and demonstrator groups were faster in correcting themselves), we found a significant effect of predation-selection treatment × demonstrator correctness in their speed of learning (treatment × demonstrator × trial: est = −0.33, SE = 0.14, *Z* = −2.37, *P* = 0.02). Despite a visual trend for predation-selected fish with an incorrect demonstrator showing a steeper learning curve (Fig. 4), pairwise contrasts of estimated marginal trends corrected for multiple comparisons were not significant for any contrast, likely due to our small sample size (detailed output in the supplemental information).

Finally, when examining learning rates over all trials, we also found an indication that treatment and demonstrator groups differed in their speed of learning (treatment × demonstrator × trial: est = −0.12, SE = 0.06, *Z* = −1.96, *P* = 0.05). However, pairwise contrasts of estimated marginal trends corrected for multiple comparisons were not significant for any contrast, likely due to our small sample size (detailed output in the supplemental information). Replicate and color groups improved their success at a similar rate (replicate × trial, est = −0.04, SE = 0.03, *Z* = −1.60, *P* = 0.11; color × trial, est = 0.001, SE = 0.05, *Z* = 0.02, *P* = 0.98). Detailed statistical outputs and learning curves can be found in the supplemental information.

## Discussion

In this study, we examined how predation selects on the use of social information in guppies by comparing how predation-selection and control fish performed in a learning task with correct or incorrect demonstrators (ie, reliable or false information). Overall success rates did not differ between treatments, nor did performance in the first trial, indicating no baseline differences in task engagement or initial copying. However, among the individuals that learned the task, only control fish showed an effect of demonstrator correctness, contrary to our prediction based on the costly information hypothesis. This suggests that predation selection decreases reliance on social information in a low-risk task. Such observed differences between the selection lines may arise either from a reduced ability to integrate social cues, diminished attention to demonstrator behavior, or a faster and more flexible adjustment to false information in the predation-selected lines. Below, we discuss the implications of these findings for our understanding of the mechanisms that underlie social information use in a predation context.

While our findings are in line with the assumption that predation pressure is linked to the evolution of social behavior and information-use tactics (Krause and Ruxton 2002; Sumpter 2006; Ward and Webster 2016), they may challenge the commonly assumed directionality, as we found no differences in learning performance in the predation-selected animals exposed to either a correct or an incorrect demonstrator. Previous research emphasizes the role of predation in selecting for larger and more cohesive groups (Seghers 1974; Magurran et al. 1992; Beauchamp 2004; Blumstein and Daniel 2005; Huizinga et al. 2009; Song et al. 2011), which in turn is thought to enhance survival by facilitating more efficient information transfer among members (Herbert-Read et al. 2015; Temizer et al. 2015). Given the established link between predation, group living, and effective information transfer, we hypothesized that predation would select for improved social information use, and thus we expected that predation-selected fish would be faster learners when exposed to a correct demonstrator but much slower when exposed to false information. Previous findings have indeed shown that animals tend to rely more heavily on social learning in contexts where risks are elevated (Kendal et al. 2004). For example, European minnows’ (*Phoxinus phoxinus*) propensity to rely on social information in a foraging patch choice is mediated by the level of predation risk: when in the presence of a dummy predator, the minnows rely more on social information even when they have reliable personal information (Webster and Laland 2008). Similarly, wild-type guppies exposed to conspecific alarm cues, indicating high-predation-risk, prioritize social information in a foraging task compared with fish exposed to a control treatment (Guigueno et al. 2025). Additionally, social learning has a large role in the development of anti-predator behaviors (Suboski et al. 1990; Mateo 1996; Mateo and Holmes 1997; Mineka and Cook 2013). For example, fathead minnows (*Pimephales promelas*) learn to exhibit anti-predator behavior in response to olfactory cues from a novel predator when these cues are received at the same time as seeing a fright response from conspecifics (Chivers and Smith 1994). However, our study suggests that the relationship between predation pressure and social information use may be more nuanced, as we do not find that predation pressure selects for individuals that prioritize social information use in low-risk contexts.

On the contrary, our results show that control fish were influenced by false information from incorrect demonstrators, while predation fish were not. This may indicate that fish from the predation lines are unable to integrate social information when learning such a foraging task, or that they prioritize personal information in this low-predation foraging context. These differences in cognitive tactics between control and predation lines may be linked to the contrasting life-history strategies that these selection lines exhibit (De Waele et al. 2025), which would be consistent with a pace-of-life syndrome (Dammhahn et al. 2018; Goulet et al. 2018): females from the predation lines show multiple traits linked to a fast life-history strategy, such as larger and earlier reproductive output (De Waele et al. 2025), which could be associated with an equally fast cognitive style where individuals make faster decisions by sampling less thoroughly and attentively (Sih and Del Giudice 2012; Goulet et al. 2018), which in our low-risk task could also equate showing low attentiveness to social information.

It is noteworthy that we did not find strong differences between both predation groups and the control fish with a correct demonstrator, either in trials to learn or in the proportion of success of the very first trial. However, our results indicate that treatment and demonstrator groups differed in their speed of learning during the first 15 trials of the task, and visual inspection of choice in the first trial and of learning rates during the first 15 trials of the task suggests that predation-selected fish with an incorrect demonstrator had a lower success at the start but recovered faster compared with the groups with a correct demonstrator. It is therefore possible that predation-selected fish exhibited a similar tendency to copy the demonstrator's choice at the start of the task but showed a better ability to handle false information compared with control fish (Mesoudi et al. 2016; Gray and Webster 2023). As responding to a false alarm represents a cost in terms of loss of energy and opportunities (Beauchamp and Ruxton 2007), and because false alarm rates are predicted to be higher under high-predation risk (Gray and Webster 2023), fish from the predation lines may have been selected to respond faster to false information. Nonetheless, we note that our sample size is small and therefore limits our ability to determine if the observed trend of predation-selected fish with an incorrect demonstrator reflects a true effect or is an artifact of low statistical power.

Additionally, it is possible that the task we subjected our fish to was not costly enough to elicit social information use in the predation group or that social learning is only used in other types of contexts. The costly information hypothesis predicts that individuals will use private information when the costs associated with doing so are low, but that they should increasingly use social information as the costs of using private information rise. In line, minnows only copy when using private information would be costly (ie under higher simulated predation risk) (Webster and Laland 2008). Similarly, high-predation guppies confronted with conflicting personal and social information about predation risk and safety prioritize social information only when personal information was uncertain (facing a novel cue) (Feyten et al. 2021). However, here we tested our fish for social information use in a low-risk environment. This environment might not have been risky enough to warrant the use of social information, with its potential for associated costs. Especially in fish with predation ancestry, switching over to using cheap, but potentially inaccurate social information might not be worth the risk when they have access to reliable personal information. A study trained groups of fish to take either a longer route or a shorter route to a feeder in a low-risk context (Laland and Williams 1998). They then gradually replaced the trained group members with untrained individuals, and found that the fish chose the energetically expensive, long route even after all founders that had been initially trained on it had been removed. Even more, the rate at which the untrained subjects that were exposed to trained individuals on the long route learned to take the short route was slower than for fish exposed to no social information at all (Laland and Williams 1998). Therefore, maladaptive information can be socially transmitted and can inhibit learning of a more suitable behavioral pattern. And while the cost of this maladaptive behavior is not too high in this particular context, in a high-predation context, this behavior could be detrimental. Therefore, the threshold to start using social information might be higher for fish with predation ancestry (Feyten et al. 2021). Therefore, it would be interesting to compare the social learning of fish from high and low-predation populations in the different levels of predator risk. This could help highlight how different types of social information use and social learning are selected for, and if the plasticity of anti-predator response learning differs between treatments. Additionally, it is possible that predation selects for different aspects of cognition, increasing their ability to learn such a task by relying on private information, and therefore eliminating the need to rely on social information.

Similarly, the propensity to rely on social learning may also be dependent on other context factors. It is possible that fish from high-predation environments need more than 1 demonstrator. In tadpoles (*Pseudacris maculata*), for example, individuals learn to show a stronger anti-predator response to salamanders (*Ambystoma tigrinum*) when the ratio of demonstrators to observers is higher (Ferrari and Chivers 2008). This has also been seen in pigeons (Lefebvre and Giraldeau 1994), rats (Beck and Galef 1989), and guppies (Lachlan et al. 1998). In high predatory contexts in particular, being alone might be comparably more dangerous. For instance, groups of fish make faster and more accurate decisions in predatory environments compared with single fish, which authors attribute to the availability of social information and reduced vigilance efforts (Ward et al. 2011). Additionally, guppies might prefer to gather personal information rather than copy group members’ decisions, up until it involves losing visual contact with the rest of the shoal; a situation that is expected to reduce the security benefits provided by swimming in a group (Kendal et al. 2004). Thus, the impact of context on social learning remains enigmatic, highlighting the need for further research to unravel the intricate relationships between predation, social learning, and survival strategies in animal populations.

In conclusion, our experiment elucidates the impact of predation on the evolution of social learning: we show that predation pressure does not necessarily facilitate the use of social information when learning a task as used here. These results suggest that while the emergence of social learning is dependent on evolutionary history regarding predation, it seems that the relationship between predation and the reliance on social learning may be more complex than previously understood. Not all types of social learning may be as advantageous in high-risk settings. This knowledge deepens our understanding of adaptive social learning evolution, and explicitly points to the need of future work to disentangle the adaptive value of different social learning forms in varied predation contexts.

## Supplementary Material

arag036_Supplementary_Data

## Data Availability

Analyses reported in this article can be reproduced using the data provided by Vila-Pouca and De Waele (2026).
