# Supplementary material for: Predation selection impacts social information use in guppies (Poecilia reticulata)
Source: Behav Ecol. 2026 Apr 22;37(3):arag036. doi: 10.1093/beheco/arag036 (PMC13102101; doi:10.1093/beheco/arag036)
Supplement: Supplementary file 1 [file arag036_supplementary_data.zip › Supplementalinformation.odt]

Supplemental information

Results

When examining choice in the very first trial of the task, to assess if fish were acting on social information and copying their demonstrators since they had no personal information about the task yet, we found no significant interaction or main effects detected of treatment and demonstrator groups. The detailed output is given in table S1.

Table S1. Outcomes of statistical model 3 examining choice in the first trial of the task. Nind = number of individuals, C+ = control fish with a correct demonstrator, C- = control fish with an incorrect demonstrator, P+ = predation fish with a correct demonstrator, P- = predation fish with an incorrect demonstrator. Significant values are given in bold.

| Factors | Estimate | Standard error | Z value | P-value |
| --- | --- | --- | --- | --- |
| Model 3: Choice in the first trial of the task (Nind=45) |
| C+, n = 11; C-, n = 11; P+, n = 11; P-, n = 12 |
| Intercept | 3.21 | 1.41 | 2.27 | 0.02 |
| Treatment | 0.32 | 0.97 | 0.33 | 0.74 |
| Colour | -2.10 | 0.92 | -2.62 | 0.01 |
| Demonstrator | 0.87 | 1.01 | 0.86 | 0.39 |
| Replicate | -0.67 | 0.54 | -1.32 | 0.19 |
| Treatment:Demonstrator | 18.13 | 2846.98 | 0.01 | 0.99 |

We also compared learning rates during the initial stages of the task (first 15 trials), to examine if certain treatment and demonstrator groups were faster in correcting themselves. We found a significant interaction treatment×demonstrator×trial indicating differences in learning rate between the treatment and demonstrator groups (table S2). However, despite a visual trend for predation-selected fish with an incorrect demonstrator showing a steeper learning curve (figure 4, main document), pairwise contrasts of estimated marginal trends corrected for multiple comparisons were not significant for any contrast, likely due to our small sample size. The detailed output is given in table S2.

Table S2. Outcomes for the probability of success for the social learning task during the first 15 trials only. Nind = number of individuals, Nobs = number of observations, C+ = control fish with a correct demonstrator, C- = control fish with an incorrect demonstrator, P+ = predation fish with a correct demonstrate or, P- = predation fish with an incorrect demonstrator. Significant values are given in bold.

| Factors | Estimate | Standard error | Z value | P-value |
| --- | --- | --- | --- | --- |
| Model 4: Probability of success across first 15 trials (Nind=45, Nobs=636) |
| C+, n = 11; C-, n = 11; P+, n = 11; P-, n = 12 |
| Intercept | 2.002 | 0.805 | 2.488 | 0.013 |
| Treatment | -2.374 | 0.409 | -5.804 | <0.001 |
| Colour | 0.058 | 0.068 | 0.848 | 0.396 |
| Demonstrator | -0.313 | 0.246 | -1.269 | 0.204 |
| Trial | -1.112 | 0.905 | -1.228 | 0.219 |
| Replicate | -1.986 | 0.872 | -2.278 | 0.023 |
| Treatment:Trial | 1.707 | 1.252 | 1.363 | 0.173 |
| Colour:Trial | 0.201 | 0.105 | 1.906 | 0.057 · |
| Demonstrator:Trial | 0.148 | 0.094 | 1.566 | 0.117 |
| Treatment:Demonstrator:Trial | -0.334 | 0.141 | -2.366 | 0.018 |
| Pairwise contrasts for the estimated marginal trends of Treatment:Demonstrator:Trial |
| P corr - C corr | -0.201 | 0.105 | -1.906 | 0.226 |
| P corr – P incorr | -0.148 | 0.094 | -1.566 | 0.398 |
| P corr - C incorr | -0.014 | 0.096 | -0.147 | 0.999 |
| C corr - P incorr | 0.053 | 0.101 | 0.523 | 0.954 |
| C corr - C incorr | 0.187 | 0.104 | 1.795 | 0.276 |
| P incorr - C incorr | 0.134 | 0.093 | 1.443 | 0.473 |

We found an indication, albeit non-significant, that treatment and demonstrator groups differed in their learning rate (treatment×demonstrator×trial: est = -0.12, se = 0.06, Z = -1.96, p = 0.05). However, pairwise contrasts of estimated marginal trends corrected for multiple comparisons were not significant for any contrast (Table S3). Fish also showed a preference for black as the correct colour (Table S3).

Figure S1. Probability of correct choice over trials of female guppies with predation (red) and control (blue) treatments, exposed to the correct demonstrator (full line), and incorrect demonstrator (dashed line). The coloured line shows predicted model outputs and shaded areas indicate 95% confidence intervals; the grey lines show individual learning curves.

Table S3. Outcomes for the probability of success for all trials of the social learning task. Nind = number of individuals, Nobs = number of observations, C+ = control fish with a correct demonstrator, C- = control fish with an incorrect demonstrator, P+ = predation fish with a correct demonstrate or, P- = predation fish with an incorrect demonstrator. Significant values are given in bold.

| Factors | Estimate | Standard error | Z value | P-value |
| --- | --- | --- | --- | --- |
| Model 5: Probability of success across all trials (Nind=45, Nobs=1139) |
| C+, n = 11; C-, n = 11; P+, n = 11; P-, n = 12 |
| Intercept | 1.469 | 0.795 | 1.848 | 0.065 |
| Treatment | -0.616 | 0.508 | -1.212 | 0.225 |
| Colour | -2.356 | 0.538 | -4.381 | < 0.001 |
| Demonstrator | -1.629 | 0.518 | -3.143 | 0.002 |
| Trial | 0.107 | 0.071 | 1.515 | 0.130 |
| Replicate | 0.053 | 0.316 | 0.167 | 0.867 |
| Treatment:Trial | 0.121 | 0.055 | 2.194 | 0.028 |
| Colour:Trial | 0.001 | 0.046 | 0.022 | 0.983 |
| Trial:Replicate | -0.042 | 0.026 | -1.602 | 0.109 |
| Demonstrator:Trial | 0.120 | 0.051 | 2.360 | 0.018 |
| Treatment:Demonstrator:Trial | -0.118 | 0.060 | -1.962 | 0.050 |
| Random effects | Variance | χ2 | Df | P-value |
| Fish identity intercept | 1.737 | 30.579 | 2 | < 0.001 |
| Fish identity × Trial slope | 0.009 | 43.935 | 2 | < 0.001 |
| Pairwise contrasts for the estimated marginal trends of Treatment:Demonstrator:Trial |
| P corr - C corr | -0.121 | 0.055 | -2.194 | 0.125 |
| P corr – P incorr | -0.120 | 0.051 | -2.360 | 0.085 |
| P corr - C incorr | -0.123 | 0.056 | -2.206 | 0.122 |
| C corr - P incorr | 0.001 | 0.063 | 0.010 | 1.000 |
| C corr - C incorr | -0.003 | 0.053 | -0.049 | 1.000 |
| P incorr - C incorr | -0.003 | 0.047 | -0.068 | 1.000 |
